# Stairway to Heaven: Evaluating Levels of Biological Organization Correlated with the Successful Ascent of Natural Waterfalls in the Hawaiian Stream Goby *Sicyopterus stimpsoni*

**DOI:** 10.1371/journal.pone.0084851

**Published:** 2013-12-27

**Authors:** Heiko L. Schoenfuss, Takashi Maie, Kristine N. Moody, Kelsey E. Lesteberg, Richard W. Blob, Tonya C. Schoenfuss

**Affiliations:** 1 Aquatic Toxicology Laboratory, Saint Cloud State University, Saint Cloud, Minnesota, United States of America; 2 Department of Biological Sciences, Clemson University, Clemson, South Carolina, United States of America; 3 Department of Food Science and Nutrition, University of Minnesota, Saint Paul, Minnesota, United States of America; Friedrich-Schiller-University Jena, Germany

## Abstract

Selective pressures generated by locomotor challenges act at the level of the individual. However, phenotypic variation among individuals that might convey a selective advantage may occur across any of multiple levels of biological organization. In this study, we test for differences in external morphology, muscle mechanical advantage, muscle fiber type and protein expression among individuals of the waterfall climbing Hawaiian fish *Sicyopterus stimpsoni* collected from sequential pools increasing in elevation within a single freshwater stream. Despite predictions from previous laboratory studies of morphological selection, few directional morphometric changes in body shape were observed at successively higher elevations. Similarly, lever arm ratios associated with the main pelvic sucker, central to climbing ability in this species, did not differ between elevations. However, among climbing muscles, the adductor pelvicus complex (largely responsible for generating pelvic suction during climbing) contained a significantly greater red muscle fiber content at upstream sites. A proteomic analysis of the adductor pelvicus revealed two-fold increases in expression levels for two respiratory chain proteins (NADH:ubiquinone reductase and cytochrome b) that are essential for aerobic respiration among individuals from successively higher elevations. Assessed collectively, these evaluations reveal phenotypic differences at some, but not all levels of biological organization that are likely the result of selective pressures experienced during climbing.

## Introduction

Nature presents a wide range of functional challenges that must be overcome by organisms if they are to survive and produce offspring for the next generation [1,2]. The selective pressures may be reflected in changes to subcellular composition, physiological capacities, structural material properties, and whole organism shape and size [3-6]. The “vertical integration of physiological processes across organizational levels within organisms” [7] has been described as one of the grand challenges facing biologists today. Testing how different levels of organization contribute to functional success can be quite challenging for a single system in its natural setting. Contributing to these challenges are the different approaches typically used to evaluate performance and variation at each level.

The amphidromous gobioid fish *Sicyopterus stimpsoni* from the Hawaiian Islands presents an excellent system for testing how different levels of biological organization might be correlated with successful functional performance in a natural setting. Hawaiian streams are typically punctuated by numerous waterfalls, many of which are several tens of meters tall [8,9]. Larval *S. stimpsoni* hatch from eggs laid in upstream freshwater habitats, and are then passively washed downstream (and over waterfalls) into the ocean by the prevailing strong currents of small Hawaiian watersheds [10-12]. Larvae grow for three to six months in the planktonic stratum of the near-shore ocean environment, then return into freshwater where they metamorphose into juveniles [13,14]. Adults may live in streams for up to five years, however, to escape predators in the estuaries and reach adult breeding habitats, juvenile *S. stimpsoni* must ascend waterfalls [8,11,12]. To climb, this species uses a slowly advancing, “inching” behavior, in which oral and pelvic suckers are alternately attached to the substrate while a portion of the body is pulled upwards [15-19]. The few juveniles that successfully ascend waterfalls find a stream environment largely devoid of predators [9]. Adult stream habitats also contain an abundance of diatoms, the primary food of these herbivorous fishes [20,21]. Indeed, adult fish will establish territories centered around feeding rocks [22] that are maintained by an individual through continuous grazing for long periods of time. These feeding rocks are also associated with mate selection [22]. 

Laboratory studies of juvenile *S. stimpsoni* have indicated several factors that might contribute to successful climbing in nature. First, selection studies on artificial waterfalls have found significant differences in external morphology between successful and unsuccessful climbers. These included patterns of selection favoring taller heads, longer attachments of the pectoral fin to the body, wider suckers, and longer dorsal and anal fins, but shorter and narrower midbody regions, shorter pectoral fins, and smaller body mass [11,12,23]. Second, the strength of suction during climbing is actively mediated by muscles running from the pelvis to the sucker. An increase in mechanical advantage of these muscles will increase adhesive ability [24,25]. In addition, compared to other goby species, the slow “inching” behavior of *S. stimpsoni* has been shown to be facilitated by an elevated proportion of red, or slow oxidative-glycolytic, fibers in trunk and tail muscles associated with climbing [26]. 

**Figure 1:**
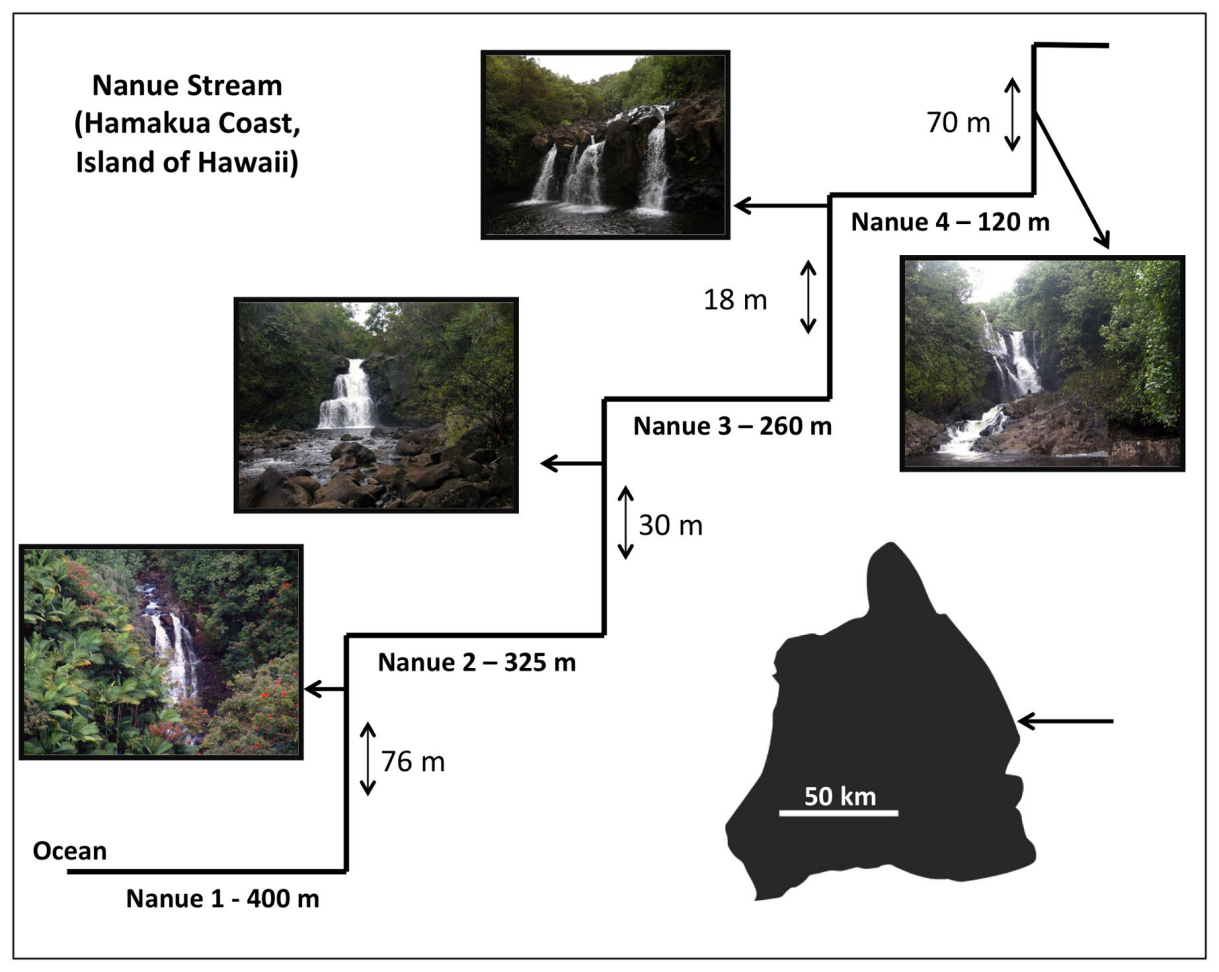
Schematic topography of Nanue stream on the Big Island of Hawai’i (Hawai’i). Arrow on the map indicates location of Nanue stream. Name and length of stream segment are indicated below each segment. Approximate heights of waterfalls are provided next to the vertical lines, with an image of each waterfall. Schematic drawing not to scale.

While this list of factors spans from whole-body morphology to muscle cell type, differences at even smaller scales of organization, such as at the level of protein expression, might also have an impact on climbing performance. In addition, while variation in several of these factors has been examined in laboratory settings, none have yet been compared across individuals with differing degrees of climbing success in nature. In this study, we test for differences across multiple levels of biological organization among individuals of the waterfall climbing fish *S. stimpsoni* from sequential pools in Nanue Stream on the Big Island of Hawai’i (Figure 1). Through these comparisons, we seek to test the overall hypothesis that successful climbing to greater heights is reflected in changes across multiple levels of biological organization.

## Materials and Methods

### Ethics Statement

Permission for access to field sites and specimens was provided by the Division of Aquatic Resources, State of Hawai’i, and coordinated by Dr. Robert Nishimoto. All collections occurred on public land and did not entail any protected species. This study was carried out in strict accordance with the recommendations in the Guide for the Care and Use of Laboratory Animals of the National Institutes of Health. The protocol was approved by the Institutional Animal Care and Use Committees of St. Cloud State University (permit numbers: 1207 and 0113) and Clemson University (permit numbers: 40061 and 2011-057). Fish were maintained in aerated coolers filled with stream water. Within 6 hours of collection, they were transferred to the research station of the Hawai’i Division of Aquatic Resources in Hilo, Hawai’i. Fish were maintained at the field station in aerated aquaria until they could be processed (usually within 48 hours) at which time they were sacrificed using MS-222. All efforts were made to minimize distress to the animals. 

### Habitat and Fish Collection

The Nanue watershed is located on the Hamakua Coast of the Big Island of Hawai’i (Hawai’i) approximately 25 km north-northwest of Hilo, HI. This 14 km^2^ watershed is characterized by a succession of tall waterfalls that begin within 400 m of the stream mouth and can be found every several hundred meters thereafter (Figure 1). Waterfall heights range from 76 m for the first waterfall to 30 m for the second, 18 m for the third, and 70 m for the fourth, dividing the stream into four study segments (Nanue 1 – estuary to Nanue 4 – most upstream). The watershed is densely forested, limiting accessibility and human impact on the stream.

Mature adult fish were collected from the four stream segments over three field seasons (2011-13). Given the small watersheds, the intensity of selection associated with waterfall climbing, and the longevity of adult *S. stimpsoni*, it was not feasible to focus our collections on juvenile fish that may have climbed waterfalls from downstream reaches more recently. Juvenile fish found above waterfalls are exceedingly rare and impossible to collect at any meaningful sample size. Collections were made while snorkeling, using bowl-shaped ‘opae nets [17]. 

### Morphometric Analysis

**Figure 2:**
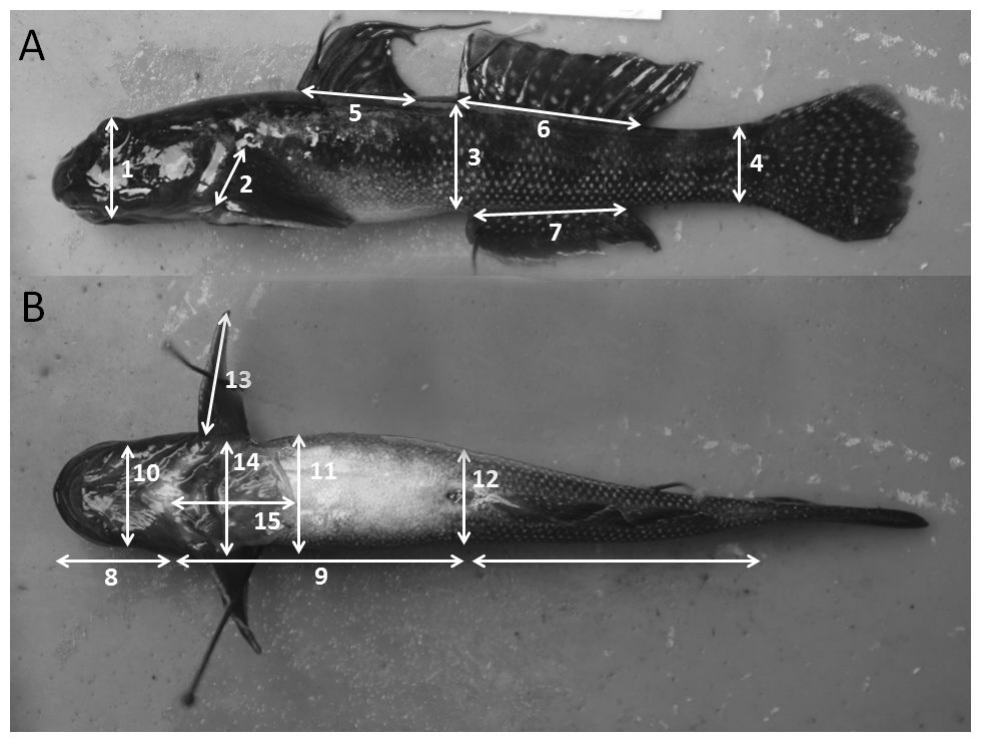
Measurements taken from *Sicyopterus stimpsoni* for morphometric analysis. Anatomical variables linked to numbers in this figure can be found in Table 1.

Samples of fish collected for morphometric analysis from each of the localities were as follows: Nanue 1, N=50; Nanue 2, N=30; Nanue 3, N=35; Nanue 4, N=21. Lateral and ventral views of all *S. stimpsoni* specimens were digitally photographed (Canon EOS Rebel with UV filter lens and 7x magnification lens), and standard length and 16 additional linear anatomical variables (Figure 2, Table 1) were measured from the photographs using ImageJ v1.44 (NIH, Washington, DC, USA, [28]). The variables selected were the same as those in previous studies of the selection imposed by waterfall climbing in juvenile *S. stimpsoni* [11,12]. These included heights of the head, midbody, and caudal peduncle; lengths of the bases of the pectoral fin, first and second dorsal fin, and anal fin; lengths of the head, trunk, tail, and pectoral fin; widths of the head and of the body at the midtrunk and anal pore; and the length and width of the pelvic sucker. To control for variation due to differences in individual size, measurements for each of the 16 variables were regressed against standard length to generate residuals. One additional variable, fineness ratio, was calculated as a further indicator of hydrodynamic performance for each specimen as the ratio of standard length to maximum height [11,12,27]. Residuals were not calculated for this variable because, as a ratio, it is already size normalized. One-way ANOVAs (with site as the main effect and d.f.=3), followed by Tukey’s post-hoc tests, were performed on the size-corrected residuals (and fineness ratio) to test for significant differences in morphological traits among the four collection sites (Nanue 1 through Nanue 4). All statistical analyses were performed in JMP (SAS Institute, Cary, NC, USA, Version 8.0.1).

**Table 1:** Predictions for patterns of change in external morphological variables for the climbing goby *Sicyopterus stimpsoni* relative to increasing elevation, with results of comparisons of variance and ANOVAs for *S. stimpsoni* across an elevational gradient of collection localities from Nanue Stream, Island of Hawai’i.

	**Expected (Blob et al. 2010) change in trait mean from low to high elevation based on selection gradients**		**Homogeneity of variance** ^2^		**ANOVA groupings of sites^3^ (sample size)**				**Observed change low to high elevation**	**Differences correspond to selection pattern predictions**
**Anatomical variable^1^**	**Direction**	**Significant?**	**Presence (F, *p*)**	**Decrease low to high elevation?**	**N1 (50)**	**N2 (30)**	**N3 (35)**	**N4 (21)**	**(F, *p*)**	
Head height (1)	Increase	Yes	Yes	N. A.	A	B	BC	C	Decrease	No
			(1.105, 0.350)						(19.980, <0.001)	
Pectoral fin base length (2)	Increase	Yes	Yes	N. A.	AB	A	BC	C	Nonmonotonic	No
			(0.460, 0.710)						(3.183, 0.0261)	
Mid-body height (3)	Decrease	No	Yes	N. A.	A	B	C	AB	Nonmonotonic	No
			(1.134, 0.338)						(16.583, <0.001)	
Caudal peduncle height (4)	Increase	No	Yes	N. A.	A	AB	B	B	Decrease	No
			(1.206, 0.310)						(3.236, 0.024)	
1^st^ dorsal fin base length (5)	Increase	No	Yes	N. A.	B	A	B	C	Nonmonotonic	No
			(0.772, 0.511)						(7.823, <0.001)	
2^nd^ dorsal fin base length (6)	Increase	No	Yes	N. A.	A	AB	AB	A	N. S.	Yes
			(0.370, 0.775)						(2.544, 0.059)	
Anal fin base length (7)	Increase	Yes	Yes	N. A.	A	A	AB	B	Decrease	No
			(1.244; 0.297)						(3.90, 0.011)	
Head length (8)	Increase	No	Yes	N. A.	B	A	B	B	Nonmonotonic	No
			(0.906, 0.440)						(5.340, 0.002)	
Trunk length (9)	Decrease	No	Yes	N. A.	AB	B	AB	A	N. S.	Yes
			(0.879, 0.454)						(2.241, 0.089)	
Head width (10)	Increase	No	No	Yes	A	AB	B	B	Decrease	No
			(7.889, <0.001)						(6.122, 0.001)	
Trunk width (11)	Increase	Yes	Yes	N. A.	A	AB	B	A	Nonmonotonic	No
			(1.944, 0.126)						(2.952, 0.035)	
Anal width (12)	Decrease	Yes	No	No	A	AB	B	A	Nonmonotonic	No
			(4.360, 0.006)						(4.137, 0.008)	
Pectoral fin length (13)	Decrease	Yes	Yes	N. A.	A	A	A	A	N. S.	No
			(0.497, 0.685)						(1.117, 0.345)	
Sucker width (14)	Increase	Yes	Yes	N. A.	A	A	A	A	N. S.	No
			(0.842, 0.473)						(1.671, 0.176)	
Sucker length (15)	Decrease	No	Yes	N. A.	A	B	B	B	Decrease	Yes^5^
			(1.004, 0.393)						(4.955, 0.003)	
Tail length (16)	Decrease	No	No	No	A	AB	AB	B	N. S.	Yes
			(4.371, 0.006)						(1.637, 0.184)	
Fineness ratio	Increase^4^	Yes	Yes	N. A.	B	B	AB	A	Increase	Yes
			(0.560, 0.642)						(3.065, 0.030)	

Note: Sample sizes for each collection locality: N1, N=50; N2, N=30; N3, N=35; N4, N=21 ^1^.See Figure 2 for illustration of these variables coded by numbers in parentheses ^**2**^.p values reported for tests of homogeneity of variance derived from Levene’s test ^3^.Alphabetical designations for the ANOVA groupings of sites follow the rank of mean values, with earlier letters indicating higher means ^4^.Selection gradients are not available for this variable (Kawano et al., 2013), so prediction was based on selection differentials ^5^.Observed pattern of change occurs in the same direction as that predicted by selection gradient but was not significant in selection experiments, whereas it was significant comparing low and high stream localities.

Choosing morphological variables that were the focus of previous selection experiments provided a basis for hypothesizing how individual traits might differ among individuals from successively higher stream segments. Selection gradients, which describe the strength of direct directional selection on a character [29], were calculated in previous studies [11,12]. These gradients indicated how specific morphological variables were subjected to significant directional selection in juveniles by the pressure of climbing a two-meter artificial waterfall. From this foundation, we hypothesized that traits previously [11,12] found to be under positive directional selection in juvenile climbers would increase with a simple, monotonic pattern among adult individuals collected from successively higher waterfalls. Conversely, traits under negative directional selection in juvenile climbers would decrease with a simple, monotonic pattern among adult individuals collected from successively higher waterfalls. We also predicted that trait variances might be lower among individuals from higher collection sites, as these individuals might have been exposed to more intense selection pressure over a greater climbing distance. Levene’s test of homogeneity of variance (implemented in JMP) was used to evaluate decreases in variable variance across collection localities. 

### Lever Arm Analysis

**Figure 3:**
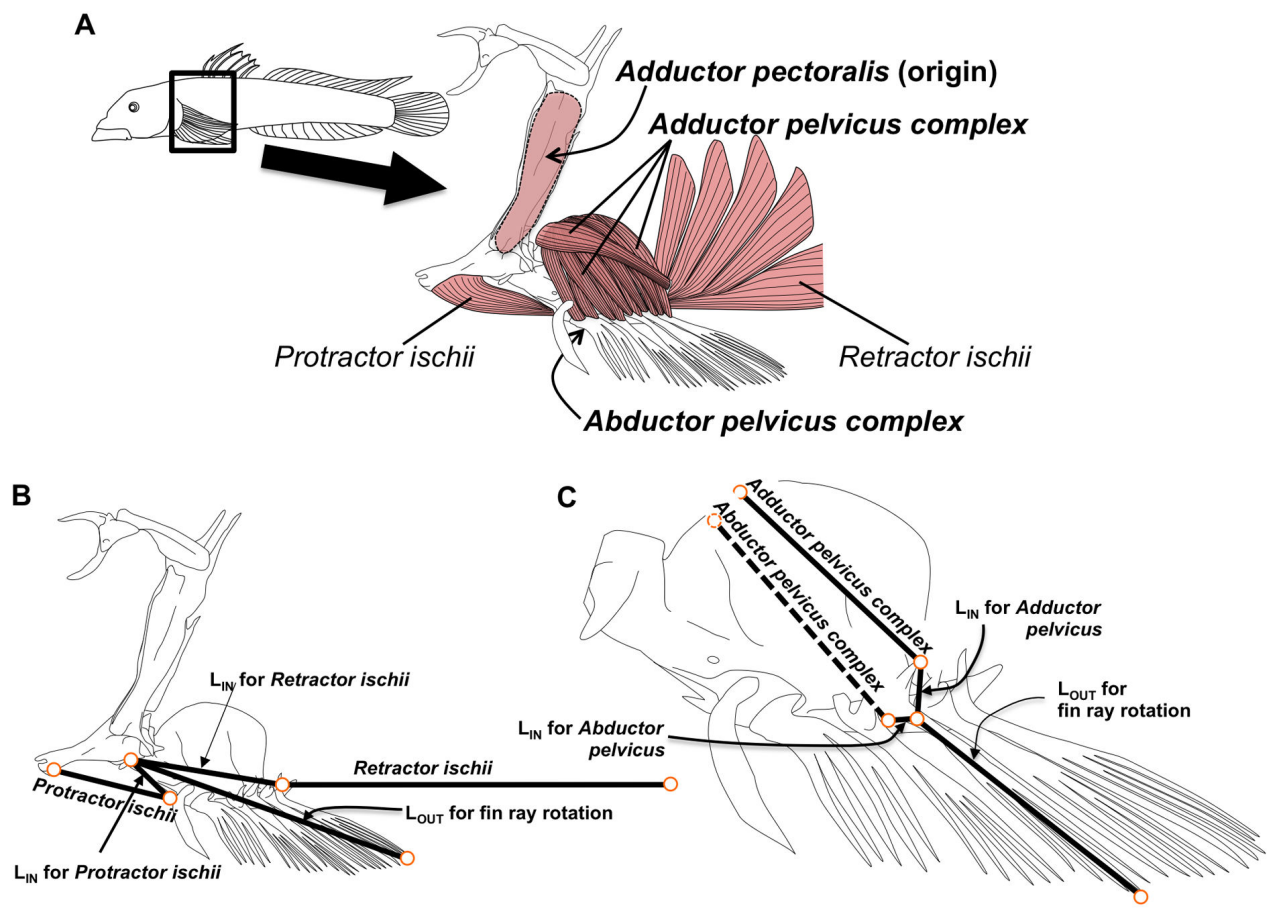
Muscles assessed for effects of climbing selection. (A) Schematic drawing of *Sicyopterus stimpsoni* (upper left) and the area of dissection (box and enlargement). Muscles and their respective in- and out-levers are indicated in the magnified drawings of the pelvic girdle (B, C). The relative position of the adductor pectoralis is indicated by the arrow near the top of the drawing. The position of the abductor pelvicus complex deep to the pelvis is indicated by the hashed line in C. Muscles for which red fiber content was established are in bold print. Drawing not to scale.

Analysis of the mechanical advantage of the lever arm system for the pelvic sucker followed previously outlined methods [25]. The pelvic muscles and skeleton of euthanized specimens from all four stream segments were dissected under a dissecting scope (SMZ 1000: Nikon Inc., Tokyo, Japan), and photographed using a digital camera (CoolPix P5100: Nikon Inc.). Samples of fish collected for lever arm analysis from each of the localities were as follows: Nanue 1, N=22; Nanue 2, N=22; Nanue 3, N=16; Nanue 4, N=10. Morphological measurements were collected from these photographs using ImageJ [28], including in-lever arm (L_IN_) and out-lever arm (L_OUT_) lengths for four muscles associated with the pelvic sucking disk (Figure 3). The protractor ischii (origin: cleithral arch; insertion: ventral face of basipterygium) and abductor pelvicus complex (origin: ventral face of the basipterygium, anterior to the insertion of the protractor ischii; insertion: heads and necks of the pelvic fin spine and rays) are both unipennate muscles involved in relaxing the grip of the pelvic sucking disk. The pennate retractor ischii (origin: the base of the anal fin spine; insertion: dorsal surface of the basipterygium) and adductor pelvicus complex (origin: dorsal face of the basipterygium, antero-dorsal to the insertion of the retractor ischii; insertion: heads and necks of the pelvic fin spine and rays) apply tension to the center of the pelvic sucking disk that strengthens its suction force. The ratios of L_IN_ to L_OUT_ for each muscle were calculated as size-normalized mechanical advantages for each individual, which were then arcsine transformed [30]. Transformed data were analyzed using ANOVAs, and Tukey post-hoc tests (JMP: SAS Institute) were used to test for differences in mechanical advantage of sucker muscles between collection sites (Nanue 1 through Nanue 4). *Sicyopterus stimpsoni* does not show ontogenetic increases in the mechanical advantage of muscles affecting sucker function as adhesive demands increase with size [25]. However, given that individuals that climb to higher elevations might require greater adhesive performance, we hypothesized that fish from higher collection sites might have greater mechanical advantages of sucker muscles. 

### Muscle Fiber Analysis

Analysis of muscle fiber types followed methods outlined in [26,31]. Fish collected for muscle fiber typing were euthanized (usually within 48 hours of capture) and the muscles to be examined were excised, placed in 98% isopentyl alcohol, and flash frozen in liquid nitrogen prior to being shipped to St. Cloud State University (SCSU), Saint Cloud, Minnesota, USA. Samples of fish collected for muscle fiber analysis from each of the localities were as follows: Nanue 1, N=10; Nanue 2, N=10; Nanue 3, N=5; Nanue 4, N=10. The small absolute size of these muscles did not allow for simultaneous analysis of tissues for histology and proteomics. However, fish for both analyses were collected concurrently during the 2012 field season. 

Fiber type analyses focused on three muscles (Figure 3). The adductor pelvicus complex was chosen because of its role in enhancing suction pressure during attachment to waterfall surfaces. The abductor pelvicus complex was included as an antagonist to the adductor pelvicus complex, which acts primarily to release suction at the end of an attachment cycle. With the greater proportion of red fibers in *S. stimpsoni* trunk and tail muscles compared to other climbing gobies [26], we hypothesized that individuals collected from higher elevations might show a greater proportion of red fibers in these muscles [24,25]. We also examined the fusiform adductor pectoralis as a reference muscle. This muscle does not play a significant role during climbing in *S. stimpsoni* and would not be expected to show differences in fiber type proportions correlated with elevation. 

Upon arrival at SCSU, muscle tissues were stored at -80°C until further processing. Each muscle tissue specimen was embedded in optimum cutting temperature medium using Cytocool II rapid freezing aerosol. Each embedded tissue specimen was cut into 8 to 17 cross-sections of 7 μm in thickness using a cryostat, and mounted on glass slides coated with tissue adhesive. This approach ensured a complete representation of the entire muscle sample in the ensuing fiber type evaluation.

To differentiate fast- and slow-twitch fibers, we performed a myofibrillar ATPase staining procedure that generates a brown precipitate in slow-twitch red muscle, following published protocols [26,31]. To avoid staining biases, we stained mixed batches of tissue samples at one time that contained an assortment of samples from several specimens and muscle types. This approach provided internal confirmation for each batch that the stain was functional, even if some tissues did not stain because they were composed entirely of fast-twitch fibers.

We counted individual red, intermediate and white muscle fibers for each section of each muscle tissue to generate the proportion of fibers that were red and intermediate. We then averaged the proportions for each specimen. Intermediate muscle fibers made up only a small proportion of total muscle fibers (<2%) and were grouped with red muscle fibers for statistical analysis (hereafter “red muscle fibers”). A small number of samples (5%) were assessed a second time to confirm the robustness of the scoring procedure. Arcsine transformation was applied to these proportional data [30]. Transformed data were analyzed by ANOVAs and Tukey post-hoc tests to examine differences in red muscle fiber proportions across muscles and sites (Prism 6.0, Graphpad, San Diego, CA). 

### Proteomics

Proteomic analysis focused on the adductor pelvicus complex because of its role in enhancing suction by the pelvic disk [24]. Based on the importance of this muscle complex in climbing and our prior understanding of the significance of red muscle to climbing performance, we hypothesized that proteins associated with red muscle physiology would exhibit greater expression at successively higher field sites. As noted above, the demands of muscle mass for histology and proteomics did not allow simultaneous application of these techniques to the same tissue samples. Excised muscle tissue samples were flash frozen, immersed in lysis solution, homogenized and frozen for shipping (N=2 fish from each of the four field sites in 2012, and N=2 fish each from Nanue 1 and Nanue 4 from collections during 2013, for a total sample of: Nanue 1, *N*=4; Nanue 2, *N*=2; Nanue 3, *N*=2; Nanue 4, *N*=4). Fish muscle extracts were shipped frozen to Bioproximity, LLC (Chantilly, VA, USA) for sample digestion and proteomic analysis. Extracts were prepared for analysis using the filter-aided sample preparation (FASP) method. After digestion with trypsin, peptides were loaded on to C_18_ Stage micropipette tips, desalted on the tips, eluted and then lyophilized, followed by fractionation on C_18_ tips using Britton-Robinson buffer at varying pH’s (see Information S1 for detailed methods). Each reaction mixture was analyzed by LC-MS/MS. 

Mass spectrometer RAW data files were converted to MGF format and were searched using X!Hunter (http://www.thegpm.org/TANDEM/index.html), cRAP (http://www.thegpm.org/crap/index.html) and X!!Tandem (http://wiki.thegpm.org/wiki/X!!Tandem). Proteins were required to have 2 or more unique peptides across the analyzed samples with E-value scores of 0.01 or less, 0.001 for X!Hunter and protein E-value scores of 0.0001 or less. We focused our interpretation on protein hits that exhibited clear trends of up- or down-regulation across the four stream segments (expression ratio between Nanue 1 and 4 consistently of log_2_ I2I over both collecting years [32]).

## Results

### External Morphology

**Figure 4:**
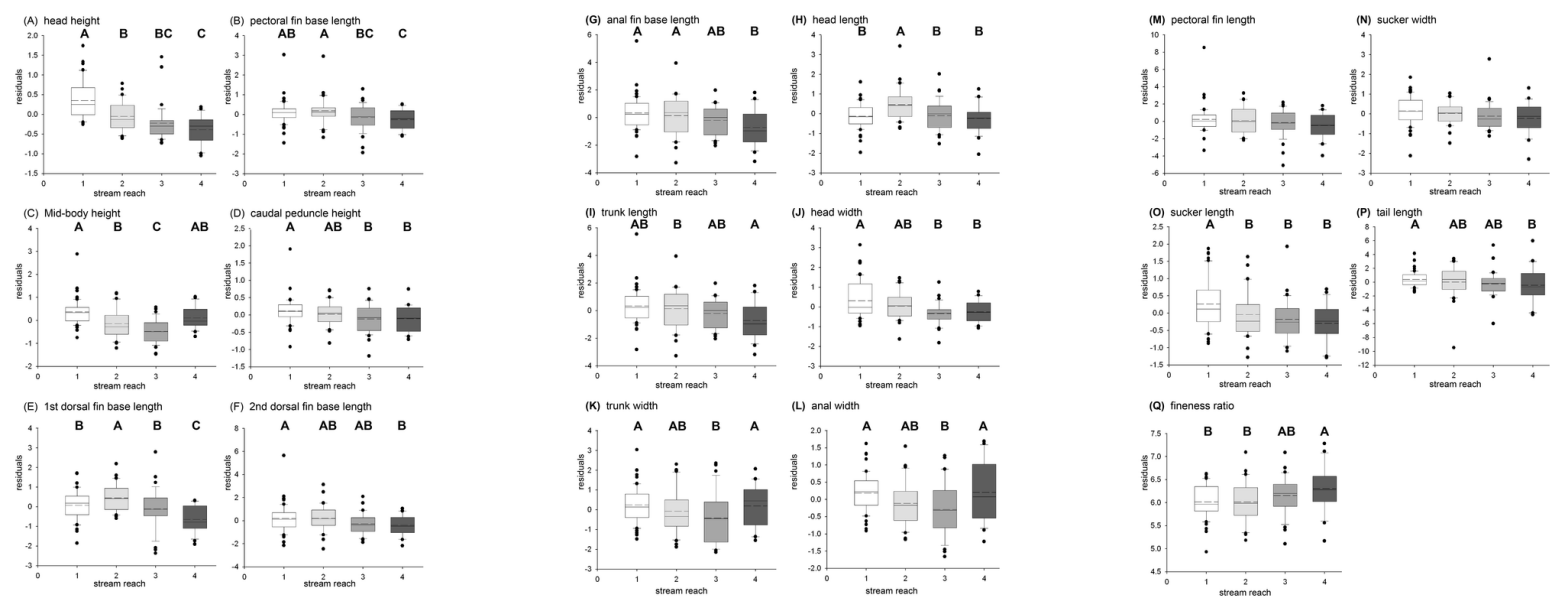
Variation in morphometric variables across successively higher elevation collecting localities. Size-corrected residuals of sixteen morphological traits (and the hydrodynamic index fineness ratio) compared among the four collection sites (Nanue 1 through 4 on the x-axis). Solid lines represent medians, dashed lines represent means, boxes indicate the 25^th^ and 75^th^ percentiles, and bars delineate 10^th^ and 90^th^ percentiles. Data points outside this range are graphed individually. Alphabetical designations for the ANOVA groupings of sites follow the rank of mean values, with earlier letters indicating higher means. Sample sizes for all graphs: Nanue 1, N=50; Nanue 2, N=30; Nanue 3, N=35; Nanue 4, N=21.

Of the 17 anatomical variables evaluated, five showed patterns that matched our predictions: second dorsal fin base length, trunk length, sucker length, tail length, and fineness ratio (Table 1, Figure 4). As we predicted, no significant differences were found in three variables (second dorsal fin base length, trunk length, tail length) between elevations. In other words, for these variables, the expectation of a non-significant pattern was met. 

Of the twelve anatomical variables that showed significant differences across the sites, five showed a consistent, monotonic decrease in magnitude from downstream to upstream sites (head height; caudal peduncle height; anal fin base length; head width; sucker length: Table 1, Figure 4), and only one showed a consistent, monotonic increase (fineness ratio). Six variables showed patterns of change across collection elevations that were not monotonic (pectoral fin base length; mid-body height; first dorsal fin base length; head length; trunk width; anal width). Of these, three showed peak values at Nanue 2, the locality immediately above the first large waterfall in the stream (pectoral fin base length; first dorsal fin base length; and head length). All three of these variables had been predicted to increase with increasing elevation based on positive selection gradients from climbing experiments (Table 1, Figure 4; [12]). However, only for pectoral fin base length was the selection gradient on which the prediction was based significant (Table 1, Figure 4; [12]). In addition, the other three variables with non-monotonic trends recorded minimum values at Nanue 3 (mid body height; trunk width; anal width). Two of these had been predicted to decrease with increasing elevation based on negative selection gradients (Table 1, Figure 4; [12]). However, only for anal width was the selection gradient on which the prediction was based significant. However, trunk width had actually been predicted (from significant selection gradients) to increase with increasing elevation (Table 1, Figure 4; [12]). 

Comparisons of variable variance across collecting localities showed only three cases in which variances were not homogeneous across the sites (head width, anal width, and tail length: Table 1, Figure 4). Of these, only head width showed a consistent decrease in variance from low elevation localities to high elevation localities.

### Sucker Muscle Mechanical Advantage

None of the four lever arm ratios assessed in this study exhibited statistically significant differences among the four study sites (Table 2). 

**Table 2:** Lever arm ratios (mean ± standard error) calculated for four muscles associated with the pelvic sucking disk in *Sicyopterus stimpsoni*, with ANOVA comparison of differences in each muscle across collecting localities.

				*Levator Ratio*			
**Nanue stream segment**	**Length (m)**	**Elevation, low-high (m)**	**Body mass (g) (N)**	**Protractor ischii**	**Retractor ischii**	**Abductor pelvicus complex**	**Adductor pelvicus complex**
1	400	0-23	2.61±0.93 (22)	0.180±0.031	0.489±0.045	0.127±0.015	0.136±0.016
2	325	99-145	3.06±1.31 (22)	0.174±0.025	0.481±0.056	0.126±0.020	0.135±0.017
3	260	175-191	3.47±1.24 (16)	0.164±0.040	0.458±0.040	0.134±0.019	0.146±0.020
4	120	209-225	4.39±1.82 (10)	0.167±0.025	0.474±0.044	0.126±0.014	0.140±0.013
				*p* = 0.235	*p* = 0.200	*p* = 0.459	*p* = 0.164
				F = 1.452	F = 1.587	F = 0.874	F = 1.748

### Muscle Fiber Typing

**Figure 5:**
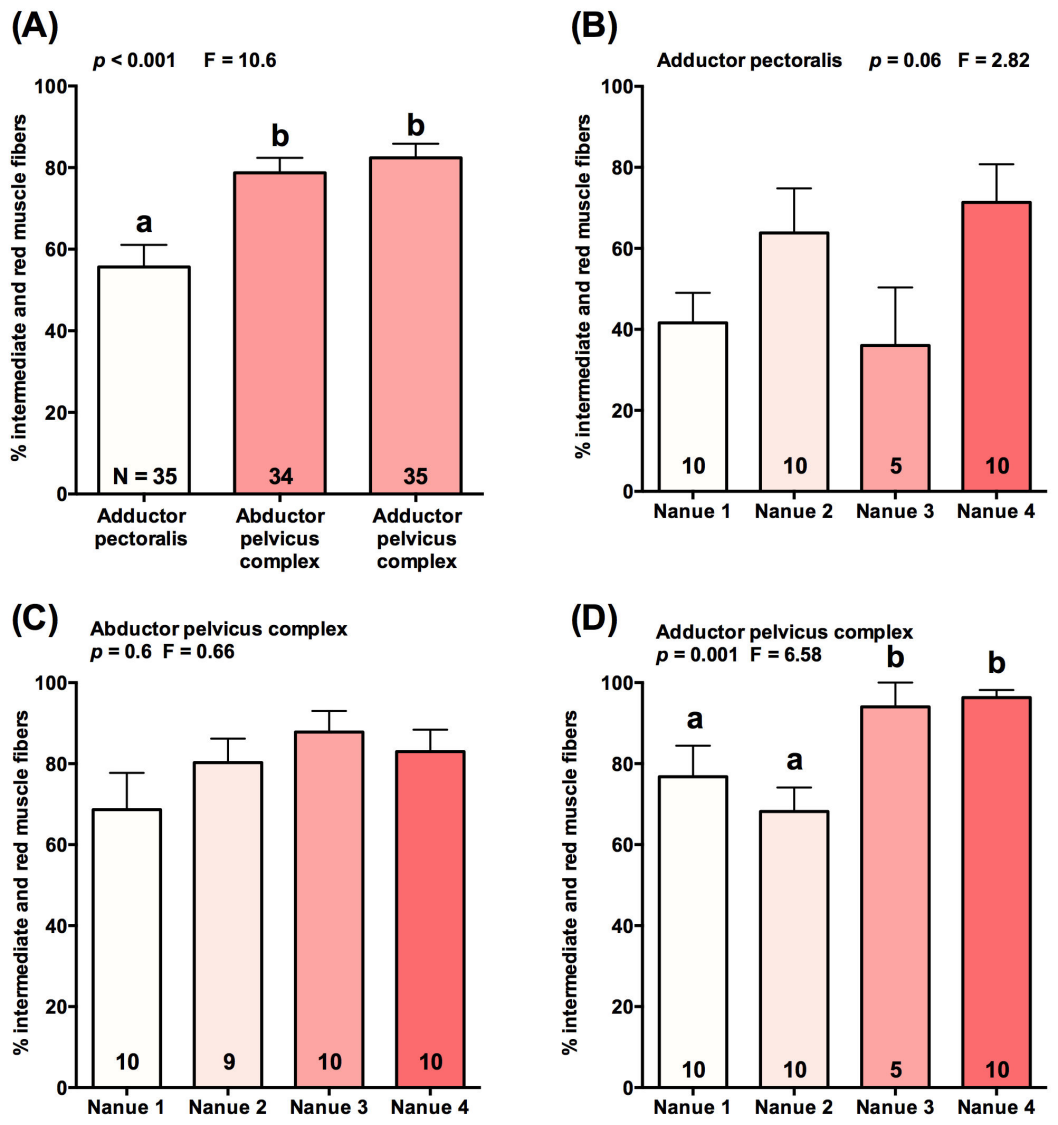
Percentage of red and intermediate fibers in three muscles in *Sicyopterus stimpsoni*. (A) Comparison of red and intermediate fiber contribution across muscle types, independent of stream segment; (B) % red and intermediate muscle fibers in adductor pectoralis, a muscle not involved in climbing; (C) % red and intermediate muscle fibers in the abductor pelvicus complex muscle; (D) % red and intermediate muscle fibers in the adductor pelvicus complex muscle. Sample size indicated in each column. Letters above columns indicate statistically significant differences between columns (p<0.05; Kruskal-Wallis with Dunn’s *post-hoc* test).

The two muscles associated with the pelvic sucking disk (abductor pelvicus complex, adductor pelvicus complex) consisted of over 80% red and intermediate muscle fibers (Figure 5A). This represented a significantly greater (*p*<0.001, F=10.58) proportion of total fibers than in the pectoral reference muscle, which averaged under 60% red and intermediate fiber content (Figure 5A). Mean red muscle fiber contributions varied by over 50% across collecting sites for the adductor pectoralis (Figure 5B) but were more consistent at only 22% variation across collecting sites for the abductor pelvicus complex (Figure 5C). In contrast, the adductor pelvicus complex exhibited a significantly greater contribution of red and intermediate muscle fibers in fish collected at Nanue 3 and 4 (*p*=0.001, F=6.58) than the two lower elevation sites. Aerobic fibers approaching 100% of muscle fibers at the two upstream sites (Figure 5D).

### Proteomics

The genetic profile of *S. stimpsoni* has not been sequenced, but database hits of peptide sequences were found to be associated with proteins in the Gobiodei. A total of 1123 protein hits were obtained through these methods. However, in many instances closely related amino-acid sequences in peptides from gobioid fishes provided multiple detections of what are homologous proteins. We focused our interpretation on the protein hits that exhibited clear trends of up- or down-regulation across elevations and provided expression ratios between Nanue 1 and 4 consistently of log_2_ I2I over both collecting years [32]. Two proteins met these criteria and were found to be associated with mitochondrial respiration (five protein hits were NADH:ubiquinone reductase, one was Cytochrome b). In both instances, tissues from the adductor pelvicus complex at Nanue 4 were found to have more than two-fold higher protein expression when compared to Nanue 1 in both collection years (Table 3). 

**Table 3:** Identified proteins in adductor pelvicus muscle tissue from *Sicyopterus stimpsoni* samples with significant differential expression collected at consecutively higher reaches of Nanue stream, Hawai’i.

			log_2_ ratio of peptide hits Nanue 4 vs 1	
**Protein**	**Peptide sequence^*1*^**	**Species^*3*^**	**2012**	**2013**
**NADH: ubiquinone reductase (H(+)-translocating) (EC=1.6.5.3)**				
F2XGN8**^*2*^**	APFDLTE	*Oplopomus oplopomus* (spinecheek goby)	**2.5**	**2.0**
	EPIRPSSSS			
	EPIRPSSSSP			
	IADGVK			
	KGPNVVGPYGLLQPIADGVK			
	MLTFIITH			
Q4KVU9	ALRAVAQK	*Giuris margaritacea* (snakehead gudgeon)	**2.1**	**2.8**
	APFDLTE			
	AVAQKILYKV			
	AVAQKILYKVSLGLILLSV			
	EPIRPSTSSPILFILAPMLALTLALILWAP			
	IADGVK			
	ILYKVSLGLIL			
	ILYKVSLGLILLSVFIFTGG			
	KGPNVVGPYGLLQPIADGVK			
	LSLPIAFAGLPPQL			
Q4KVY3	APFDLTE	*Leptophilypnus panamensis*	**2.1**	**2.6**
	EPIRPSTSSPLLFILAPMLALTLALILWAP			
	IADGVK			
	IYIVPVLLAVAFLTLLER			
	KGPNVVGPYGLLQPIADGVK			
	YALIGALRAVPQTISYEV			
Q94WB2	IADGVK	*Ptereleotris microlepis* (blue gudgeon)	**2.1**	**2.0**
	KGPNVVGPYGLLQPIADGVK			
	QTISYEV			
Q94WF2	AGLPPQT	*Awaous guamensis* (scribbled goby)	**2.8**	**2.0**
	APFDLTE			
	HLSLSVALAGLPPQT			
	IADGVK			
	KGPNVVGPYGLLQPIADGVK			
	LSVALAGLPPQT			
**Cytochrome b**				
Q4KW45	DVNFGWLIR	*Gobiomorus maculatus*	**2.5**	**2.0**
	ETWNVGVVL			
	FTPANPL			
	IANDALVDLPAPSN			

***^1^***Peptide sequences identified in muscle tissue in *S. stimpsoni*; **^*2*^**UniProtKB protein accession number (www.uniprot.org); ***^3^*** species that the peptide sequences matched

## Discussion

This study evaluated aspects of phenotypic variation that have the potential to contribute to successful functional performance during waterfall climbing. Through these comparisons, we sought to test the overall hypothesis that successful climbing to greater heights would be reflected in traits across multiple levels of biological organization. In contrast to our a priori expectations, the variation correlated with successful penetration to higher elevations was concentrated at particular levels of organization. The nature of correlations between climbing to high elevations and morphological features were particularly unexpected for two reasons. First, external body shape exhibited patterns that differed from expectations, and second, lever arm ratios failed to show significant correlations. Furthermore, several features of external body shape showed maximum or minimum values above intermediate waterfalls rather than the highest waterfall. This finding suggests that fish possessing advantageous values of some traits might climb only to a certain point, rather than as high as possible, before establishing territory in a stream.

### Correlations between External Body Morphology and Upstream Climbing

Previous laboratory selection analyses on juvenile *S. stimpsoni* showed several aspects of external body morphology to be under significant directional selection due to the demands of waterfall climbing [11,12,23]. However, only one of the 17 morphological variables we examined (head width) showed a consistent decrease in variance moving from low to high localities, as might be expected for a trait under strong selection in the field (Table 1, Figure 4). In addition, very few of our external morphological variables showed differences between elevations that matched our expectations. Of the five variables that did match expectations from selection analyses, three did so because both selection analyses and elevational comparisons failed to show significant differences. In addition, one variable (sucker length) showed a significant decrease in field data that matched a non-significant trend from selection analyses (Table 1, Figure 4). Only one variable, fineness ratio, showed a matching, significant pattern between lab and field data, showing significant increases in mean from low to high locations in both (Figure 4). This variable is a hydrodynamic index for which increases (up to a value of 10) indicate closer approach to an optimal value for reducing drag [27]. Such a capacity could be highly advantageous for fishes ascending increasingly higher waterfalls. The strength of the correlation between selection studies and field data for patterns in fineness ratio may reflect the single best aspect of body shape that contributes to upstream climbing and residence above high waterfalls. 

Although monotonic directional changes in trait means did not frequently match expectations, four additional variables did show significant monotonic decreases in value as elevation increased: head height, caudal peduncle height, anal fin base length, and head width (Table 1, Figure 4). Decreases in the height and breadth of body components would be expected to reduce drag in gobies. In addition, reduction of the anal fin might allow closer adherence of the body to the substrate, placing the fish more within the boundary layer of flow where velocity is slower and easier to resist [11]. Several factors may contribute to the fact that such patterns were found among fish collected in the field, but not during selection studies. First, due primarily to sample size limitations, selection studies were conducted on juvenile fish [11,12,23], but our field samples collected adult fish. Selection might act differently on fish of different sizes [33]. In addition, other aspects of drag reducing body shapes may contribute functional advantages during swimming and in stream residence for adults, which may not have emerged from analyses of climbing performance in juveniles. 

In fact, the most common pattern for external morphological variables was for a peak (or low) at an intermediate elevation locality (Table 1, Figure 4). Three variables showed peak values at Nanue 2, immediately above the first waterfall in the stream (pectoral fin base length, first dorsal fin base length, and head length), and three showed minimum values at Nanue 3 (midbody height, trunk width and anal width). Because adult *S. stimpsoni* live for multiple years, it is possible that the adults collected in this study belong to multiple age cohorts, which may have been subject to different patterns of selection. It is also possible that the climbers with the most hydrodynamic body shape do not necessarily climb the furthest upstream. The individuals that ascend the first waterfall the soonest after a flash flood event may be able to establish a territory [10], forcing subsequently arriving fish to climb further. Similarly, competition for feeding rocks, territories, and mates may force juveniles to continue climbing beyond the pool above the first waterfall. The functional significance of the traits showing maxima at Nanue 2 is not immediately apparent. In contrast, lower values of the three traits showing minima at Nanue 3 all could reduce drag [11] and potentially allow for earlier climbing and establishment of a territory. Ecological factors responsible for the cessation of climbing, or continued climbing past the first waterfall of a stream, will require further study.

### Lever Arm Ratios of Skeletal Elements Associated with Climbing

No differences in mechanical advantage of sucker muscles across fish from different collection elevations were found. This result was unexpected, as it contrasted with functional predictions based on previous studies of the scaling of adhesive forces in the pelvic sucker [24]. Adhesive performance of the pelvic sucker in *S. stimpsoni* was previously found to increase allometrically despite isometric growth of pelvic sucker attachment area, suggesting that other skeletal factors (such as mechanical advantage) contribute disproportionally to adhesive performance [24]. Therefore, it is surprising that changes in mechanical advantage do not contribute to climbing further upstream in *S. stimpsoni*, given that such changes could improve performance with limited addition of metabolically demanding tissue. However, other skeletal systems of gobies also have shown limited variation in muscle lever systems within species [25]. It is possible that constraints on these systems in gobies might promote selection at more fundamental (i.e., cellular or molecular) levels of organization. 

### Elevation and Red Muscle Fiber Proportions

In contrast to lever arm ratios, significant patterns were identified in comparisons of muscle fiber types across localities, with a greater proportion of red fibers found in the adductor pelvicus complex of fish from sites 3 and 4 compared with sites 1 and 2 (Figure 5). This increase in red fibers would convey a greater capacity for sustained, aerobic contraction of a muscle, contributing to adhesive force in higher-climbing fishes. This is supported by the significant difference in red muscle fiber contribution between sites for the adductor pelvicus complex. No changes were found in muscles which either are of little functional significance in climbing (adductor pectoralis) or, in the case of the abductor pelvicus complex, perform a function with a fixed demand (i.e., breaking the seal of the sucker at the end of the suction cycle). Taken together, the lack of differentiation of red muscle fiber contribution in two fixed demand muscles, contrasted with increased red muscle fiber contribution in the primary climbing muscle, suggest that the differences observed are related to climbing proficiency. Similar to observations of peaks in body shape selection, the most pronounced difference in red muscle fiber content in the adductor pelvicus complex was found between intermediate Nanue sites 2 and 3. These findings provide further evidence that functional advantages may enable juveniles to cease climbing once they are able to establish a territory.

Increases in red muscle fiber content in the locomotor muscle of *S. stimpsoni* are contrary to observations made in other fish species that ascend steep stream gradients. Studies of the streaked prochilod (*Prochilodus lineatus*) ascending a fish ladder in Brazil identified increased white muscle fiber proportions as a hallmark of fish that had successfully passed this artificial selective barrier [34]. Similarly, pink salmon (*Onchorhynchus gorbuscha*) that successfully navigated fish passages in British Columbia, Canada contained higher white muscle lactate concentrations after completion of their climb, suggesting anaerobic exercise and a strong contribution of white muscle fibers to locomotion [35]. These authors highlighted the great sensitivity of muscle tissue to exercise-related physiological changes [35]. The reliance of many fish on white muscle to ascend steep stream gradients also matches well with observations of “powerburst” waterfall climbing behavior in the only two other species of Hawaiian gobioid fishes (*Awaous guamensis, Lentipes concolor*) capable of ascending waterfalls [15,26]. It remains to be studied whether waterfall climbing imposes a selective pressure favoring higher white fiber content in the climbing muscles of successful powerburst climbers in Hawai’i, and indeed across numerous oceanic islands where many other species of amphidromous gobioid fishes can be found [36]. The “inching” climbing style, with slow and continuous cycles of sucker attachment is unique to the genus *Sicyopterus* and, thus, may have produced very different physiological targets of selection than found in most gobioid fishes capable of climbing waterfalls.

### Changes in Protein Expression Associated with Changes in Elevation

Quantification of protein expression aids in identifying subtle changes in physiology related to the selective pressure of waterfall climbing. The proteomic analysis of the adductor pelvicus complex revealed the enhanced expression of proteins involved in aerobic oxidation that increased relative to fish from the Nanue 1 site with each further upstream locality. This effect was identified in both years during which fish were collected for this study. NADH:ubiquinone reductase is part of complex I of the respiratory chain found in the mitochondria of eukaryotes, and is an essential component of aerobic respiration [37]. Proteomics data are consistent with the higher proportion of red muscle fibers in the adductor pelvicus complex at upstream Nanue sites, suggesting higher rates of aerobic respiration in fishes at higher elevations. Additionally, Cytochrome b is part of the respiratory chain complex III, which is also found in the mitochondria of eukaryotes [38] and is expressed more in upstream Nanue sites when compared to site 1. Increases of NADH-d associated with mitochondrial aerobic respiration were also found in muscle tissues during a laboratory endurance study of zebrafish (*Brachydanio rerio*) [39] and provide further evidence that muscle endurance is a crucial component of successful waterfall climbing in *S. stimpsoni*. 

As in our analyses of body morphology, the use of mature fish for proteomics entails some limitations. Proteomics is a tool frequently used to identify rapid changes in organismal physiology following an environmental challenge [32,40]. Many of the changes in protein expression are likely of short duration [41] and are likely not represented in the tissues analyzed for this study. However, since proteomics represents a direct measure of gene expression [42], physiological processes that have been favored by the selection incurred through successful waterfall climbing may still be manifested in the protein expression profile of adult fish. Studies utilizing younger fish and large sample sizes will be needed to further decipher the effects of selective pressures incurred after waterfall climbing from those incurred during climbing.

### Do Different Levels of Biological Organization Correlate with Functional Performance – How Far is a Successful Climb?

This investigation supported some specific hypotheses that were based on data from prior experiments, but refuted others. Laboratory studies of selection [11,12,23] can provide a reasonable basis for expectations about variation in morphological traits across environmental gradients in nature, but a large range of factors can confound such correspondences. Nonetheless, the strongest correspondence we found was for a trait (fineness ratio) with the most direct known impact on functional performance [33]. It is encouraging that strong signals can be recovered despite the potential for confounding factors between lab and field studies. The lack of correlation between climbing performance and lever arm ratios suggests that instances of enhanced suction performance [24] could, instead, result primarily from a physiological level of organization (i.e., enhanced red muscle fiber contribution). 

The consistent enhanced expression of two proteins associated with aerobic respiration is consistent with the increased contribution of red muscle fibers to the adductor pelvicus complex in fish at higher elevations. These two respiratory proteins are potential targets of selection, though additional proteins yet to be identified may contribute as well. The increased levels of expression of these two proteins with increased elevation may reflect selection on proteins that are upstream in the biochemical pathways, but that we were not able to detect with the protein alignments used here from other species. These results provide preliminary evidence for the selective pressures associated with waterfall climbing and resultant changes manifested at the genotypic level. Given our small sample size and single time point collections, the results of the protein expression analysis provide directions for future studies. 

Given the maximum (or minimum) values of several variables at intermediate collecting localities, it appears that climbing furthest may not directly correspond to the greatest climbing success. Climbing just one waterfall is sufficient for *S. stimpsoni* to escape predators (which cannot climb [8,9]) and find suitable habitats. Indeed, climbing further upstream may imply the inability of some fish to compete for suitable feeding rocks, territories, and/or mates. Further study of the ecology of these fishes will be required to quantify the relative contributions of these and other factors that may drive climbing beyond the first waterfall in *S. stimpsoni*.

Most broadly, the results of this study indicate the span of variation across levels of biological organization that can contribute to successful functional performance. Identifying and exploring divergent results from laboratory and field studies will generate new hypotheses to elucidate how selection is integrated vertically across levels of biological organization. By evaluating possible targets of natural selection related to waterfall climbing at different levels of biological organization, this study provides a framework to connect effects observed at the phenotype with possible variation reflecting the genetic basis of characters in a natural environment. 

## Supporting Information

Materials and Methods S1 **Detailed proteomics methods.**
(DOCX)
