# Supplementary material for: Stairway to Heaven: Evaluating Levels of Biological Organization Correlated with the Successful Ascent of Natural Waterfalls in the Hawaiian Stream Goby Sicyopterus stimpsoni
Source: PLoS One. 2013 Dec 27;8(12):e84851. doi: 10.1371/journal.pone.0084851 (PMC3873996; doi:10.1371/journal.pone.0084851)
Supplement: Materials and Methods S1 — Detailed proteomics methods. (DOCX) [file pone.0084851.s001.docx]

**SUPPORTING INFORMATION – S1**

**Stairway to Heaven: Evaluating Levels of Biological Organization Correlated with the Successful Ascent of Natural Waterfalls in the Hawaiian Stream Goby *Sicyopterus stimpsoni***

Heiko L. Schoenfuss^1^*, Takashi Maie^2^, Kristine N. Moody^2^, Kelsey E. Lesteberg^1^, Richard W. Blob^2^, and Tonya C. Schoenfuss^3^

^1^Aquatic Toxicology Laboratory, Saint Cloud State University, Saint Cloud, Minnesota, USA

^2^Department of Biological Sciences, Clemson University, Clemson, South Carolina, USA

^3^Department of Food Science and Nutrition, University of Minnesota, Saint Paul, Minnesota, USA

**Materials & Methods**

Detailed Proteomics Methods

Excised muscle tissue was quickly frozen with either liquid nitrogen or dry-ice, then immersed in lysis solution (8M urea, 0.2% SDS, 0.5M TEAB and 4 mM TCEP) at a concentration of 30 μL 246 solution/mg tissue (N=2 fish from each of the four field sites in 2012, and N=2 fish each from Nanue 1 and Nanue 4 from collections during 2013, for a total sample of: Nanue 1, *N*=4; Nanue 2, *N*=2; Nanue 3, *N*=2; Nanue 4, *N*=4). Tissue was homogenized in the buffer by either sonication or with a glass mortar and Teflon pestle, and frozen for shipping. Samples were stored at -80°C. Fish muscle extracts were shipped frozen to Bioproximity, LLC (Chantilly, VA) for sample digestion and proteomic analysis.

*FASP Method*. Samples were brought to 2% SDS, 50 mM Tris-HCl, pH 7.6, 10 mM DTT and heated at 95°C for 10 min. Samples were transferred to a 30 k Amicon molecular weight cut-off device (Millipore, Billerica, MA) and centrifuged at 13 k g for 30 min. The remaining sample was buffer exchanged with 6 M urea, 100 mM Tris-HCl, pH 7.6, then alkylated with 55 mM iodoacetamide. Concentrations were measured using a Qubit fluorometer (Invitrogen, Grand Island, NY). Trypsin was added at a 1:40 enzyme to substrate ratio and the sample incubated overnight on a heat block at 37°C. The device was centrifuged and the filtrate collected.

*Peptide Desalting and Fractionation*. Digested peptides were desalted using C_18_ Stage micropipette tips. Briefly, for each sample a C_18_ Stage tip was activated with methanol, and then conditioned with 60% acetonitrile with 0.5% acetic acid followed by 5% acetonitrile with 0.5% acetic acid. Samples were loaded onto the tips and desalted with 0.5% acetic acid. Peptides were eluted with 60% acetonitrile, 0.5% acetic acid and lyophilized in a SpeedVac (Thermo Savant, Thermo Fisher Scientific, Waltham, MA) to dryness, approximately 2 hrs. Peptides were fractionated by dissolving samples in Britton-Robinson buffer pH 10 and loaded on to C_18_ Stage tips. Flow-through was collected. Subsequent fractions were taken by eluting peptides with Britton Robinson buffers at pH 8, 6, 5, 4 and 3.2 and capturing with C_18_ Stage tips. Peptides were eluted from the C_18_ Stage tips and dried as described above.

*Liquid Chromatography-Tandem Mass Spectrometry*. Each reaction mixture was analyzed by LC-MS/MS. LC was performed on an Easy nanoLC II HPLC system (Thermo Fisher). Mobile phase A was 94.5% MilliQ water, 5% acetonitrile, 0.5% acetic acid. Mobile phase B was 80% acetonitrile, 19.5% MilliQ water, 0.5% acetic acid. The 120 min LC gradient ran from 2% B to 35% B over 100 min, with the remaining time used for sample loading and column regeneration. Samples were loaded to a 2 cm x 100 um I.D. trap column positioned on a Rheodyne actuated valve (IDEX Health and Science, Oak Harbor, WA). The column was 13 cm x 100 um I.D. fused silica with a pulled tip emitter. Both trap and analytical columns were packed with 3.5 um C_18_ resin (Zorbax SB, Agilent, Santa Clara, CA). The LC was interfaced to a dual pressure linear ion trap mass spectrometer (LTQ Velos, Thermo Fisher) via nano-electrospray ionization. An electrospray voltage of 1.8 kV was applied to a pre-column tee. The mass spectrometer was programmed to acquire, by data-dependent acquisition, tandem mass spectra from the top 15 ions in the full scan from 400 - 1400 m/z. Dynamic exclusion was set to 30 s.

*Mass Spec Data Analyis*. Mass spectrometer RAW data files were converted to MGF format using msconvert. Briefly, all searches required strict tryptic cleavage, 0 or 1 missed cleavages, fixed modification of cysteine alkylation, variable modification of methionine oxidation and expectation value scores of 0.01 or lower. MGF files were searched using X!Hunter (http://www.thegpm.org/TANDEM/index.html) against the latest library available at the time on The Global Proteome Machine (GPM) (http://www.thegpm.org). Other searches used the cRAP (http://www.thegpm.org/crap/index.html) contaminant library from GPM and libraries constructed from the latest Ensembl release available at the time (http://www.ensembl.org/index.html). MGF files were searched using X!!Tandem (http://wiki.thegpm.org/wiki/X!!Tandem) using both the native and k-score scoring algorithms and by Open Mass Spectrometry Search Algorithm (OMSSA) (http://pubchem.ncbi.nlm.nih.gov/omssa/). All searches were performed on Amazon Web Services-based cluster compute instances using the Proteome Cluster Interface (http://www.bioproximity.com/proteome-cluster.html). XML output files were parsed and non-redundant protein sets determined using MassSieve (http://www.ncbi.nlm.nih.gov/staff/slottad/MassSieve/). Proteins were required to have 2 or more unique peptides across the analyzed samples with E-value scores of 0.01 or less, 0.001 for X!Hunter and protein E-value scores of 0.0001 or less. We considered differences to be significant if log_2_ of the ratio between Nanue 1 and 4 was ≥ 2 or ≤ -2 in both sampling years.
